# The alterations in the extracellular matrix composition guide the repair of damaged liver tissue

**DOI:** 10.1038/srep27398

**Published:** 2016-06-06

**Authors:** Mariliis Klaas, Triin Kangur, Janeli Viil, Kristina Mäemets-Allas, Ave Minajeva, Krista Vadi, Mikk Antsov, Natalia Lapidus, Martin Järvekülg, Viljar Jaks

**Affiliations:** 1 Department of Cell Biology, Institute of Molecular and Cell Biology, University of Tartu, Estonia; 2 Laboratory of Physics of Nano Structures, Institute of Physics, University of Tartu, Estonia; 3 Department of Pathological Anatomy and Forensic Medicine, Department of Biomedicine, Institute of Biomedicine and Translational Medicine, University of Tartu, Estonia; 4 Department of Pathology, Clinic of Diagnostics, West Tallinn Central Hospital, Estonia; 5 Department of Biosciences, Karolinska Institutet, Stockholm, Sweden

## Abstract

While the cellular mechanisms of liver regeneration have been thoroughly studied, the role of extracellular matrix (ECM) in liver regeneration is still poorly understood. We utilized a proteomics-based approach to identify the shifts in ECM composition after CCl_4_ or DDC treatment and studied their effect on the proliferation of liver cells by combining biophysical and cell culture methods. We identified notable alterations in the ECM structural components (eg collagens I, IV, V, fibronectin, elastin) as well as in non-structural proteins (eg olfactomedin-4, thrombospondin-4, armadillo repeat-containing x-linked protein 2 (Armcx2)). Comparable alterations in ECM composition were seen in damaged human livers. The increase in collagen content and decrease in elastic fibers resulted in rearrangement and increased stiffness of damaged liver ECM. Interestingly, the alterations in ECM components were nonhomogenous and differed between periportal and pericentral areas and thus our experiments demonstrated the differential ability of selected ECM components to regulate the proliferation of hepatocytes and biliary cells. We define for the first time the alterations in the ECM composition of livers recovering from damage and present functional evidence for a coordinated ECM remodelling that ensures an efficient restoration of liver tissue.

Liver is an organ with a remarkable regenerative capacity. Liver regeneration in response to an injury involves restoration of functional liver tissue through proliferation of both mature as well as stem/progenitor cells and remodelling of the extracellular matrix (ECM)^1^. Furthermore, such life-threatening pathological conditions as liver cirrhosis and liver cancer are accompanied by aberrant changes in ECM structure and composition^2^.

The ECM has been traditionally considered an inert cell growth substrate; however, during the last decade the knowledge about the biological role of the ECM has greatly developed. At present day, the ECM is recognized as a dynamic structure, which is composed of a variety of proteins and other macromolecules and provides a supportive scaffold that actively regulates the biological functions of the cells, at least partly by interaction with specific cell surface molecules^3^. For example, integrins play a major part in transmitting the information from ECM to cells and by synergizing with other cell surface molecules like growth factor receptors regulate migration, proliferation, angiogenesis, inflammation or apoptosis^4^,^5^. Therefore, changes in the ECM composition alter cell signalling in liver and facilitate either normal regeneration or pave the way for liver diseases^1^. It should be noted that as liver ECM is produced by the cellular components of liver, the ECM remodelling taking place during regeneration and pathological processes results from changes in protein synthesis pattern and pericellular proteolytic activity of liver parenchymal and stromal cells but also invading inflammatory cells^6^.

Collagens and fibronectin are the main structural constituents of ECM. Type I and III collagens (col1 and col3) are highly expressed in liver capsule, portal stroma, Disse’s space and fibroid tissue^7^,^8^. Type IV collagen (col4) and laminins make up the basal lamina of the blood vessels and bile ducts^8^. Type V collagen (col5) forms thin fibers located in the centre of thick col1 and col3 fibrils. It is notable that more than five-fold increase in collagen deposition has been found in fibrotic livers compared to a healthy organ^9^. Fibronectin is a glycoprotein that can be found in the liver capsule, portal stroma and Disse’s space. In normal adult tissues its levels are modest but increase rapidly during tissue regeneration^10^. Recent studies showed that the absence of fibronectin in liver leads to more extensive liver cirrhosis induced by liver damage and was accompanied by increased liver stiffness and disorganized collagen network^11^.

The aim of the current work was to identify the changes in the liver ECM composition during liver regeneration and to study the potential mechanisms by which these regulate the proliferative properties of liver cells. To achieve this we utilized two well-established mouse models of toxic liver injury coupled with tissue decellularization and mass spectrometry. We identified prominent changes in the content of main structural components of the liver ECM as well as identified multiple alterations in the amounts of minor ECM constituents known to regulate tissue regeneration and development. Similar alterations in ECM were found to be present in injured human livers. Scanning electron microscopy (SEM) analysis revealed the loss of elastic fibers and microfibrils in damaged livers and the resulting increase in liver ECM stiffness was identified with atomic force microscopy (AFM). Interestingly, we found that the expression of a number of ECM proteins differed in pericentral and periportal areas. Since the analysis of the growth promoting properties of these proteins *in vitro* showed selective enhancement of the proliferative potential of either hepatocytes and or non-hepatocyte cells encompassing the biliary cell compartment we outlined a model where the identified changes in ECM composition ensure coordinated restoration of liver tissue.

## Materials and Methods

### Mice

Wild type CBA/J age- and sex-matched mice at 8–12 weeks of age were used in experiments. Acute liver injury was induced by intraperitoneal injection of CCl_4_ (1 ml/kg) in sunflower oil and mice were sacrificed after 48 hours. Alternatively, mice were fed with 0.1% diethoxycarbonyl dihydrocollidine- (DDC-) supplemented diet for 2 weeks. The procedures involving animals were carried out in accordance to the guidelines approved by the Commission of Laboratory Animal Licenses at the Estonian Ministry of Agriculture (license number 25).

### Liver decellularization

Mice were anesthetized by intaperitoneal injection of diazepam and hypnorm (1:1, 200 μl). Hepatic portal vein was cannulated and liver perfusion was carried out at 37 °C and at the speed of 5 ml/min. Mouse liver was perfused sequentially with heparinized phosphate buffered saline (PBS) (12.5 U heparin/ml, AppliChem, Darmstadt, Germany) for 10 min, 1% SDS and 1% Triton X-100 for 2 h each followed by perfusion with water for 30 min and PBS for 2 h. To evaluate the decellularization efficiency fresh mouse liver and decellularized liver samples were minced. DNA content was quantified after proteinase K digestion using a NanoDrop 2000 spectrophotometer (Thermo Scientific, Wilmington, DE). All decellularized liver samples contained less than 200 ng/mg DNA.

### Mass-spectrometry analysis

Decellularized liver samples (n = 3) were homogenized in lysis buffer containing 7 M urea, 4% CHAPS, 10 mM DTT and 40 mM Tris-HCl (pH 7.5). Proteins were purified by methanol/chloroform protein precipitation. Next, proteins were reduced, alkylated and digested by Lys-C protease and trypsin. The peptides were purified by C18 StageTips and separated on Agilent 1200 series nano-LC with in-house packed (3 μm ReproSil-Pur C18AQ particles) 15 cm 75 μm ID emitter-columns. Separated peptides were eluted to a LTQ Orbitrap XL mass-spectrometer operating with a top-5 MS/MS strategy. Raw data were processed with MaxQuant 1.4.0.8 software package and UniProt (www.uniprot.org) database using the tryptic digestion rule. See Supplementary Methods for further details.

### Cryosectioning

Mouse tissue samples were frozen in Tissue Tek O.C.T compound (Sakura Finetek, Tokyo, Japan). 7-μm cryostat sections were mounted on Superfrost Plus slides (Knittel Glass, Braunschweig, Germany) and stored at −80 °C until further analysis.

### Scanning electron microscopy (SEM)

Decellularized liver samples from normal, CCl_4_- or DDC-damaged mouse livers were prepared for SEM as described above. The samples were dehydrated through alcohol gradient starting at 50% ethanol up to 100% ethanol. Samples were dried using a Leica EM CPD300 critical point dryer (Leica Microsystems GmbH, Wetzlar, Germany) and covered with a 5 nm layer of gold using a Quorum Technologies Polaron SC7640 Sputter Coater. Fibrous structure of liver matrix was investigated by scanning electron microscope FEI Nova NanoSEM 450 (FEI, Eindhoven, the Netherlands).

### Tissue elasticity measurements

To characterize the mechanical properties of decellularized liver samples the atomic force microscope (AFM) based technique was applied^12^. Indentation was conducted with an AFM tip (SCM-PIT, Bruker, Camarillo, CA; ~25 nm tip diameter, normal stiffness approx. 3 N/m). For each sample, six force-displacement curves were measured at different sample locations. On the basis of each force-displacement curve, the indentation modulus was calculated by applying the Hertzian contact mechanics model (for further details see Supplementary Methods).

### Liver cell isolation

Mouse liver cells were isolated using a previously described method^13^ with modifications. Briefly, the mouse livers were perfused with 0.5 mM EGTA and 0.35 mg/ml type II collagenase, 1 M CaCl_2,_ both in Krebs-Ringer buffer. Cells were released into ice-cold William’s E medium (Life Technologies) by mechanically disrupting the digested liver. To isolate hepatocytes the cells were repeatedly centrifuged at 50 g for 2 min until no new pellet was formed. The supernatant containing non-hepatocyte fraction cells (NHC) were centrifuged at 300 g for 10 min and resuspended in full growth medium (William’s E medium supplemented with 5% fetal bovine serum, 50 ng/ml hepatocyte growth factor, 50 ng/ml epithelial growth factor, 1 mM sodium pyruvate, 1 μM dexamethasone, 10 μl/ml insulin-transferrin-selenium-x and 36 μl/ml Thawing/Plating cocktail A). The hepatocyte pellet was washed with William’s E medium and suspended in the full growth medium (for details see Supplementary Methods).

### Liver cell culture on coated tissue culture plates

The NHC and hepatocyte fractions were seeded on 35 mm Petri dishes coated with fibronectin, col1 or col4 (Corning Incorporated, Kennebunk, ME) in complete growth medium at 2 × 10^5^ cells per plate. Cells were cultured for 7 days with media changes every second day. For experiments utilizing Armcx2-, Thbs4- or Olfm4-conditioned media, the NHC and hepatocytes were grown on col1-coated plates in complete growth medium and on day 3 the conditioned medium collected from Armcx2-, Thbs4- or Olfm4-transfected HEK293 cells was added (ratio 1:5). Next, the cells were cultured for further 5 days. For details on cloning and conditioned media preparation see Supplementary Methods.

### Human liver samples

Human liver samples were obtained from 5 patients with mild liver damage, 5 patients with moderate liver damage, and 5 patients with severe liver damage. The severity of liver fibrotic damage was estimated according to the Scheuer’s adapted scoring system^14^. Samples of control liver tissue were obtained as contaminating liver tissue in excised gallbladder samples of two cholecystectomy patients. Written informed consent was obtained from the research subjects. The experiments were carried out in accordance with the study protocol approved by the Tallinn Medical Research Ethics Committee (permit no 820). Liver samples were fixed in 10% neutral buffered formalin overnight prior to paraffin embedding using standard protocol. 2-μm sections were cut on microtome and mounted on Superfrost Plus slides (Thermo Scientific, Braunschweig, Germany). Deparaffinization and heat-induced antigen retrieval in 10 mM sodium citrate buffer, pH 6.0, were performed before immunofluorescence staining and microscopy.

### Immunofluorescence analysis

Frozen tissue sections or cultured liver cells were fixed with 4% paraformaldehyde, permeabilized with 0.1% Triton X-100 in PBS for 10 min and blocked with 5% normal donkey serum (Jackson ImmunoResearch, West Grove, PA) for 60 min. Sections were stained with primary antibodies overnight at 4 °C (see Supplementary Table S1 for complete primary antibody list). Sections were counterstained with DAPI (0.1 μg/ml, Sigma-Aldrich, St. Louis, MO) and mounted with fluorescent mounting medium (DAKO, Glostrup, Denmark). Images were captured with Olympus IX81 CellR microscope (Olympus Corporation, Hamburg, Germany) equipped with Hamamatsu Orca ER (Hamamatsu Photonics, Herrsching am Ammersee, Germany) camera and 10x or 40x objective and processed using Hokawo 2.1 software (Hamamatsu Photonics).

### Data analysis and statistics

Mass-spectrometry data was analysed in R (http://www.r-project.org/). Statistical significance was determined by 2-way ANOVA followed by 2-tailed Student’s *t* test. *P* < 0.05 was considered significant. Other statistical analyses and graphs were generated using Graph-Pad Prism version 4.0 (GraphPad Software, San Diego, CA).

## Results

### The liver damage-induced cell proliferation is accompanied by significant changes in liver ECM composition

**Figure 1:**
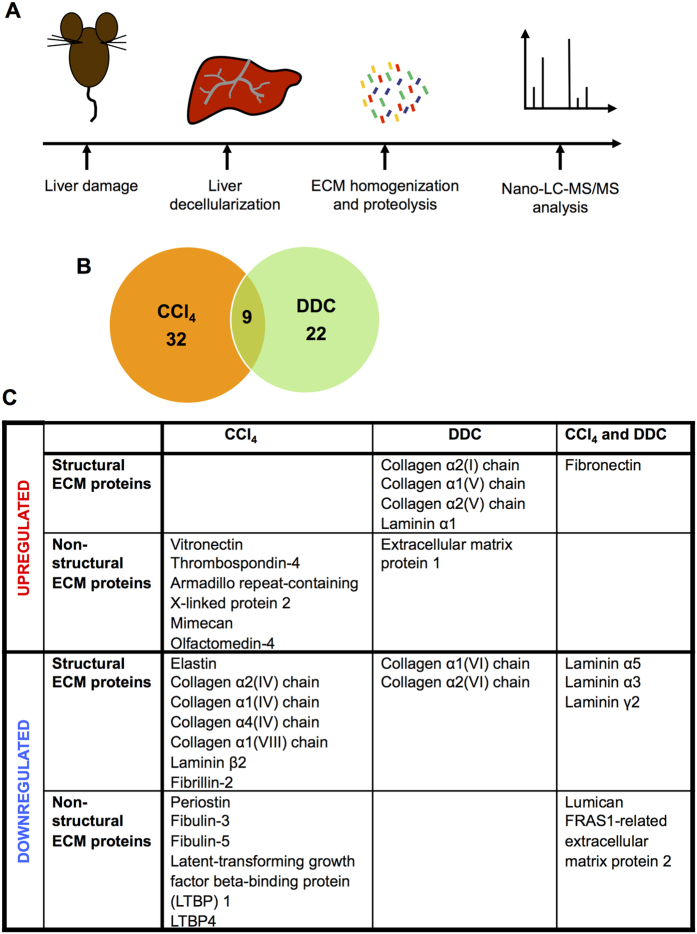
Proteomics analysis of liver ECM. (**A**) Study scheme. Groups of mice (n = 3) were subjected to intraperitoneal injection of CCl_4_ (48 h) or DDC diet (2 wk). At specified time points the livers were decellularized followed by proteomics analysis by nano-LC-MS/MS. (**B**) Summary of the results of proteomics studies. Number of proteins changed in CCl_4_- and DDC-induced liver damage and number of proteins changed simultaneously in both damages are shown. (**C**) ECM proteins up- or downregulated in damaged liver samples.

To characterize the changes in liver ECM composition in response to liver damage we injected the mice with CCl_4_ or subjected to DDC-diet^15^,^16^. We collected liver samples at timepoints with the highest proliferative activity during the course of tissue repair: 48 h after CCl_4_ administration or 2 weeks after the start of DDC diet (Supplementary Fig. S2). Subsequently, liver was decellularized and the protein extracts prepared from mouse liver ECM were analysed using nano-LC-MS/MS (Fig. 1A). Livers from untreated control animals were used for reference.

Overall, 1383 proteins were detected in decellularized liver samples. In addition to the known ECM constituents, secreted factors and cell membrane proteins a few intracellular proteins were detected as contaminants that presumably originated from cell lysis during the decellularization procedure (Supplementary Table S3). We found that the levels of 63 proteins (4.6%) were significantly altered in injured livers (Fig. 1B). 22 proteins were upregulated and 19 downregulated in response to CCl_4_ treatment; 19 proteins were upregulated and 12 downregulated in response to DDC treatment. The levels of 2 proteins were upregulated and those of 7 proteins were downregulated in response to both treatments. In general, we found prominent changes in the main structural ECM components (collagens type I, IV, V, VI, VIII; laminins α1, α5, α3, β2, γ2; elastin and fibronectin) as well as identified multiple changes in minor ECM constituents, which have been shown to play a role in tissue remodelling or regeneration (eg vitronectin, olfactomedin-4 (Olfm4), mimecan, thrombospondin-4 (Thbs4), armadillo repeat-containing x-linked protein 2 (Armcx2))^17^,^18^,^19^,^20^,^21^ (Fig. 1C).

As expected, the nature of the liver damage defined the specific molecular alterations occurring in the liver ECM. The treatment with CCl_4_, which primarily targets hepatocytes, induced the deposition of vitronectin. This was accompanied by a decrease in elastin content and downregulation of fibrillin-2, the component of microfibrils^22^, col4 and EGF-containing fibulin-like extracellular matrix protein 1 (fibulin-3), a secreted protein that regulates cell proliferation and migration^23^ (Fig. 1C). DDC treatment, which primarily affects bile ducts, resulted in upregulation of col1 and col5, which form thick collagen fibrils^24^, laminin α1 and downregulation of col6, a component of structurally unique microfibrils^25^ (Fig. 1C).

Interestingly, both liver injuries resulted in the deposition of fibronectin and downregulation of laminins α3, α5, γ2, the main constituents of basal lamina (Fig. 1C)^26^. In addition, we detected reduced expression of lumican, a small proteoglycan involved in collagen fibril assembly^27^ and FRAS1-related extracellular matrix protein 2, which is involved in the maintenance of epithelial integrity^28^. The relatively small number of altered proteins and consequently the low number of proteins altered simultaneously in both treatments was a result of the large variation between the samples. For example, col1 was generally upregulated in CCl_4_-treated livers; however, the result was statistically insignificant.

**Figure 2:**
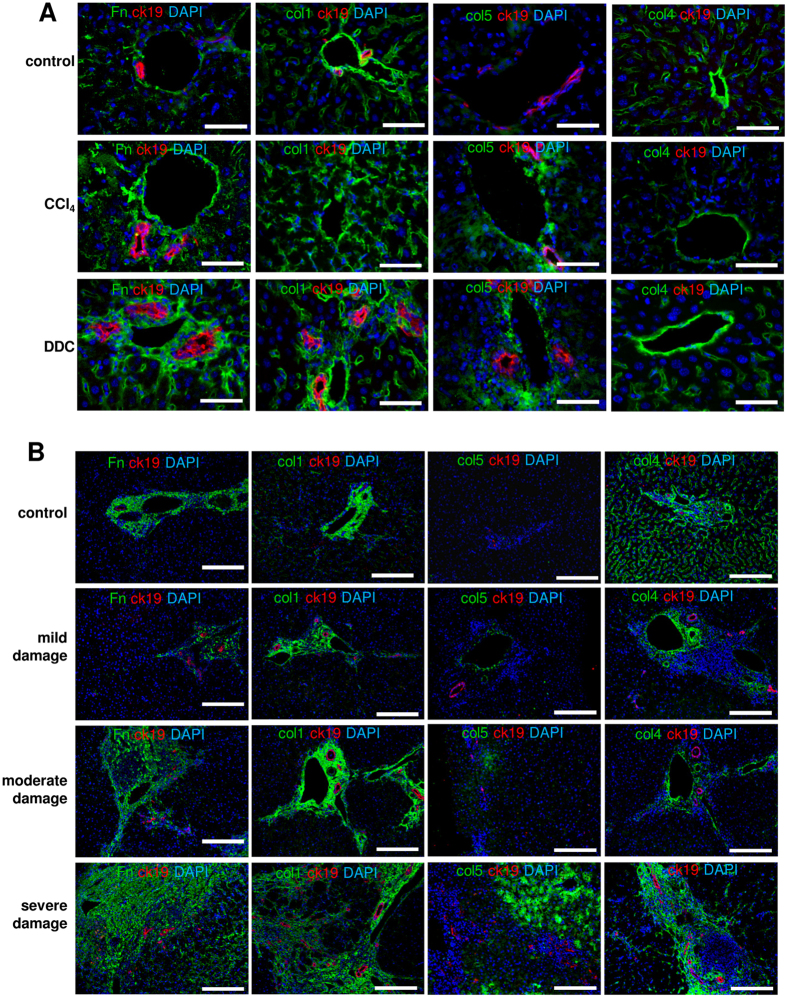
Immunofluorescence microscopy analysis of structural ECM components altered in damaged mouse (A, n = 4) and human (B, n = 5) livers. Fn-fibronectin. Scale bars 50 μm (**A**), 200 μm (**B**).

Next, we used immunofluorescence microscopy to verify our proteomics data and studied the expression and localization of the structural ECM components that were altered in the livers of CCl_4_- or DDC-treated mice. Samples obtained from damaged mouse livers and human livers with different tissue damage grades were studied in parallel (Fig. 2B, Supplementary Fig. S3). In damaged mouse livers fibronectin accumulated around portal areas and sinusoids (Fig. 2A). Correspondingly, an increased fibronectin deposition that positively correlated with the tissue damage grade was seen in human livers (Fig. 2B, Supplementary Fig. S3). In CCl_4_- and DDC-damaged mouse livers an increased col1 deposition was detected in the pericentral and periportal areas respectively, thus, labeling the sites of primary tissue damage (Fig. 2A, Supplementary Fig. S4). In human samples col1 deposition was detected in pericentral areas and was more intensive in severely damaged livers (Fig. 2B). Similarly, col5 accumulated in portal and pericentral areas in damaged mouse livers and in human livers with severe tissue damage (Fig. 2A,B). Col4 expression was reduced in pericentral sinusoids of both CCl_4_-treated mouse and damaged human liver samples (Fig. 2A,B). In contrast, an increased col4 signal was present in the portal areas of DDC-treated mouse livers (Fig. 2A). While in normal mouse livers elastin was readily detectable, only a thin elastin lining around the portal vein was observed in CCl_4_-damaged samples (Supplementary Fig. S5A). In damaged human liver samples, however, elastin expression localized in fibrotic septs and was even increased (Supplementary Fig. S5B).

### The alterations in liver ECM composition are coupled with the changes in tissue microarchitecture and elasticity

**Figure 3:**
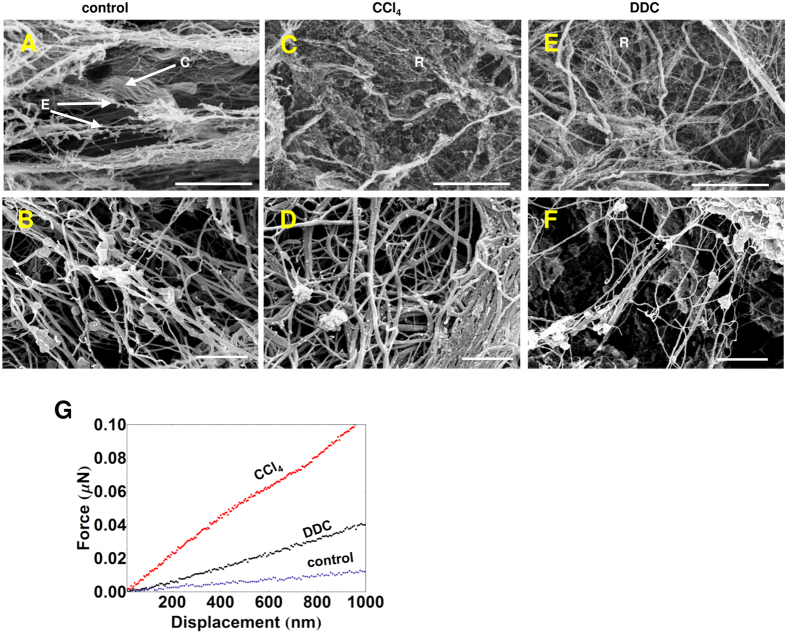
Alterations in the microstructure of damaged livers. SEM images of control (**A,B**), CCl_4_- (**C,D**) and DDC-treated (**E,F**) decellularized livers. E-elastic fibers, C-collagen fibers and R-reticular fibers. Scale bars: 10 μm (top panels), 500 nm (bottom panels), n = 3. (**G**) The average force-displacement curves of decellularized liver matrices. The median elastic stiffness for control, CCl_4_- and DDC-treated livers: 0.18 ± 0.02 MPa, 0.9 ± 0.3 MPa and0.35 ± 0.03 MPa, respectively.

The proteomics analysis indicated the increase in specific collagen forms and decrease in elastin, microfibril-forming fibrillins and col6 (Fig. 1C). As such changes are expected to alter the microarchitecture of liver tissue we subjected the decellularized liver samples to SEM analysis. Elastic fibers appear as cobwebbed cords entangled with microfibrils in the normal liver ECM (Fig. 3A,B)^29^. In the ECM of CCl_4_-treated livers, however, we observed a significant reduction in such fibers, (Fig. 3C,D). Whilst in normal liver ECM wavy cords or tape shape fibers of organized collagen fibrils prevail (Fig. 3A,B), in CCl_4_- and DDC-treated livers an increase in disorganized, thin, branching reticular collagen fibers was seen (Fig. 3C–F)^29^.

The decrease in the elastic fibers and subsequent changes in liver microarchitecture prompted for possible alterations in the mechanical properties of liver ECM. Therefore, we took advantage of AFM indentation for direct measurement of the stiffness of decellularized liver samples (Fig. 3G). As anticipated, we identified a remarkable increase in stiffness of CCl_4_- and to a lesser extent in DDC-treated liver samples.

### The liver ECM structural components differ in their ability to sustain the growth of liver cells

**Figure 4:**
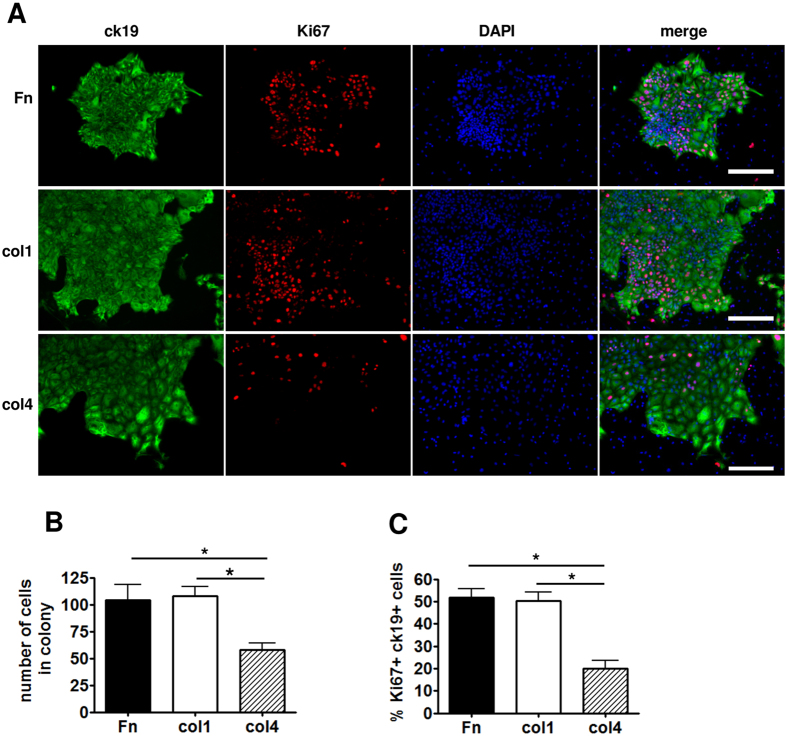
Non-hepatocyte cell growth assay on dishes coated with fibronectin (Fn), col1 or col4. (**A**) Colonies stained with anti-ck19 and -Ki67 antibodies. Scale bars: 200 μm. (**B**) Statistical analysis of the average number of cells in a colony on differently coated plates. (**C**) Percentage of proliferating ck19 + cells. Bars show the average of 3 independent experiments ± SD. Two-way ANOVA tests were used to assess differences between groups, *indicates a statistically significant (P < 0.01) difference.

**Figure 5:**
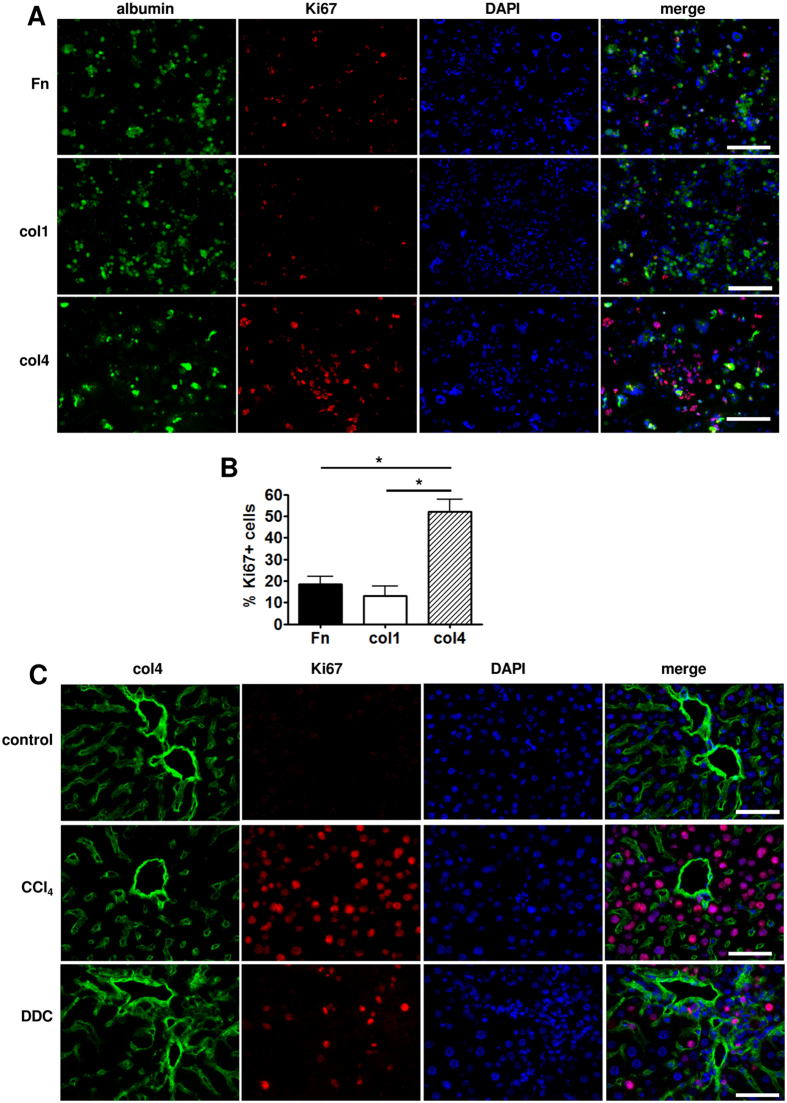
Hepatocyte fraction cell growth assay on dishes coated with fibronectin (Fn), col1 or col4. (**A**) Cells stained with anti-albumin and -Ki67 antibodies. Scale bars are 200 μm. (**B**) Percentage of proliferating cells on dishes coated with either Fn, col1 or col4. Bars show the average of 3 independent experiments ± SD. *indicates a statistically significant (P < 0.01) difference. (**C**) Immunofluorescence microscopy analysis of col4 and Ki67 in portal areas of damaged mouse livers (n = 4). Scale bars: 50 μm.

Next we hypothesized that the main structural components altered in damaged liver ECMs may differ in their ability to support the growth of liver cells. To study the elementary effects of these structural ECM proteins, the hepatocyte and non-hepatocyte cell (NHC) fractions isolated from normal mouse livers were cultivated on cell culture dishes coated with fibronectin, col1 or col4. We found that culturing NHCs, which encompasses also biliary epithelial cells, on col1 and fibronectin that were upregulated in both CCl_4_- and DDC-treated livers resulted in larger and more proliferative ck19-positive colonies of cholangiocyte-like cells compared to culturing of NHCs on col4 (Fig. 4A–C). In contrast, hepatocytes were most proliferative on col4, which was downregulated in the pericentral areas of CCl_4_-treated livers (Fig. 5A,B, Supplementary Fig. S6). To solve this apparent contradiction we co-stained normal and injured mouse liver sections with antibodies recognizing Ki67 and col4. Interestingly, in both DDC- and CCl_4_-treated livers, the proliferative hepatocytes were localized predominantly in the space surrounding portal areas, where the col4 expression was retained at higher level (Fig. 5C) suggesting that col4 promotes hepatocyte proliferation both *in vitro* and *in vivo*.

### The non-structural liver ECM components as potential regulators of liver repair

The liver damage-related changes of selected non-structural ECM components identified by mass spectrometry were verified by immunofluorescence microscopy. We focused on vitronectin, Olfm4, Thbs4 and Armcx2 expression that were upregulated in CCl_4_-treated livers due to their known involvement in tissue regeneration or development^17^,^18^,^19^.

**Figure 6:**
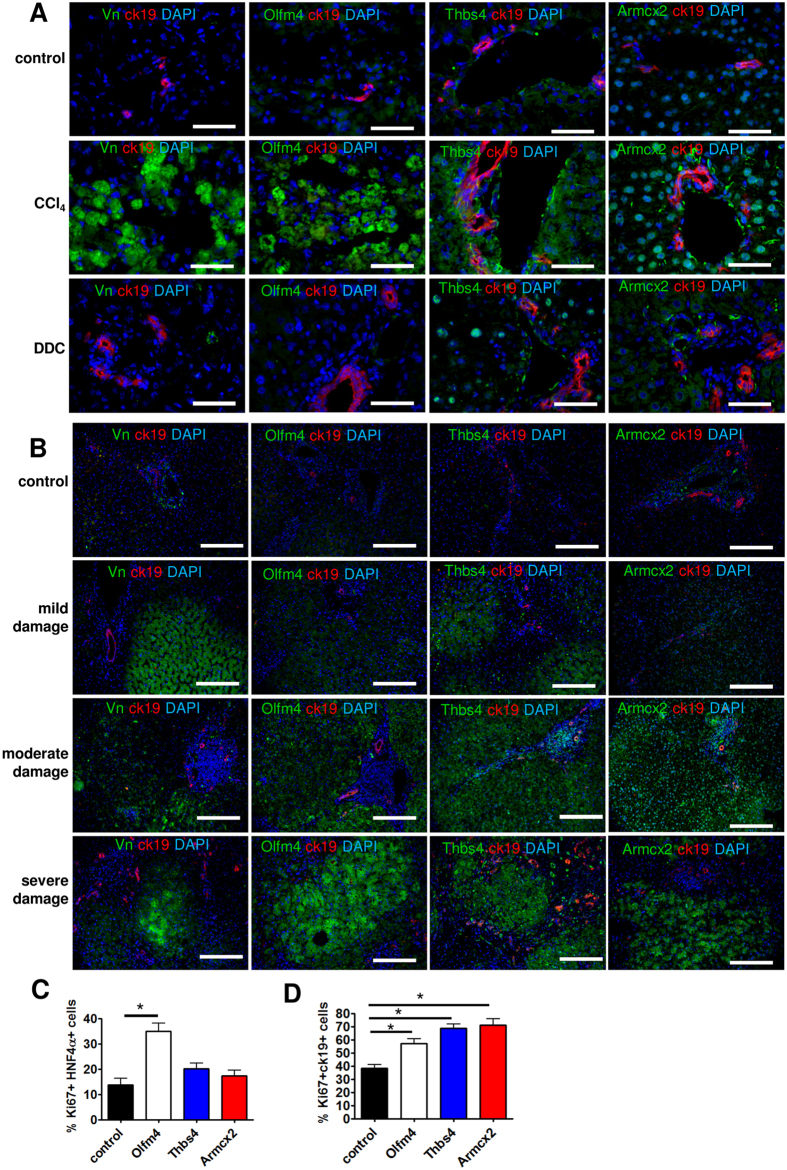
Immunofluorescence microscopy analysis of non-structural ECM components altered in mouse (A, n = 4) and human (B, n = 5) livers. Scale bars: 50 μm (**A**), 200 μm (**B**). Vn-vitronectin. Hepatocyte (**C**) and NHC (**D**) growth assays on dishes coated with col1 and added HEK293-conditioned media containing recombinant Olfm4, Thbs4 or Armcx2. Media from sham-transfected cells was used as control. The percentage of proliferating cells ± SD is shown (n = 3). *indicates a statistically significant (P < 0.01) difference.

Vitronectin localized in and around of a large population of hepatocytes in the vicinity of the central vein in CCl_4_-damaged mouse livers (Fig. 6A, Supplementary Fig. S7A). The pericentral hepatocytes are the first to undergo necrosis in toxic liver injury due to their location at the site of the metabolic activation of several toxins including CCl_4_^30^. Consequently, the pericentral hepatocytes did not proliferate and remained Ki67-negatve in response to liver damage unlike the periportal hepatocytes, which were predominantly positive for Ki67 (Supplementary Fig. S7A). In DDC-treated mouse livers there was no significant increase of vitronectin expression.

In human samples, vitronectin also accumulated preferentially in lobular hepatocytes (Fig. 6B) and its expression was more prominent in severely damaged livers. Olfm4 expression was largely overlapping with that of vitronectin in injured and normal mouse liver samples. Strong Olfm4 signal was detected in pericentral hepatocytes in CCl_4_-damaged mouse livers (Fig. 6A, Supplementary Fig. S7B). No significant expression was detected in DDC-damaged or control livers. Similarly, in human samples, Olfm4 expression was increased in lobule centres and the signal intensity correlated positively with the degree of liver damage (Fig. 6B). Thbs4 expression was significantly increased in and around the cells surrounding portal area of CCl_4_-damaged mouse livers (Fig. 6A). In DDC-damaged mouse livers the expression of Thbs4 was only modestly increased and was detected in the nuclei of hepatocytes. In human samples, Thbs4 accumulated in lobular hepatocytes and portal myofibroblasts; the expression was highest in moderately and severely damaged livers (Fig. 6B). Armcx2 showed a strong expression in the hepatocytes and in tissue structures surrounding bile ducts of CCl_4_-damaged mouse livers (Fig. 6A). In DDC-damaged mouse livers Armcx2 expression pattern was similar, albeit less intensive (Fig. 6A). In human samples, the level of Armcx2 was increased in the lobular hepatocytes and this correlated positively with the degree of liver damage (Fig. 6B).

To study the role of these factors in regulating the growth of liver cells we treated the hepatocyte and NHC cultures with Olfm4-, Thbs4- or Armcx2-conditioned media (Fig. 6C, Supplementary Fig. S8). Interestingly, when Olfm4 stimulated the growth of hepatocytes (Fig. 6C), Thbs4 and Armcx2 were most efficient in stimulating the proliferation of NHC (Fig. 6D).

The collected results demonstrate that the expression of studied regulatory ECM components largely follows a similar pattern in damaged human and mouse livers.

## Discussion

While cellular mechanisms of liver regeneration have been extensively investigated^31^, the role of liver microenvironment and ECM in this process is still poorly understood. In the current work we, for the first time, identified the shifts in ECM composition during liver regeneration using a high throughput proteomics approach and studied the role of these alterations on the proliferative potential of liver cells.

We found that the level of 4.6% of structural and non-structural proteins were altered in ECMs of mouse livers subjected to tissue damage. However, only 14% of these were altered in both CCl_4_ and DDC-induced liver damage underlining the dissimilar nature of these liver injuries. For instance, structural collagens col1 and col5 were upregulated in DDC-induced acute liver injury while col4 was downregulated after CCl4 treatment. Both CCl_4_- and DDC-damage caused the upregulation of fibronectin. In general, similar alterations have been previously found in cirrhotic livers^32^, however, in contrast to our results, an increase in col4 and elastin has been described in patients with liver fibrosis and cirrhosis^33^,^34^.

**Figure 7:**
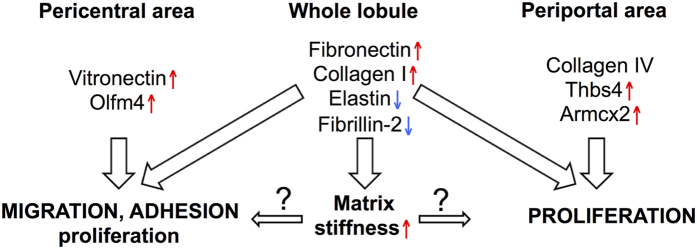
The alterations in ECM constitution regulate liver repair. See Discussion for closer explanation.

The changes in major structural ECM components were translated into significantly altered tissue microarchitecture and elasticity as the ECMs of damaged livers were significantly stiffer compared to control liver ECMs. However, in different liver damage models these changes were apparently caused by distinct mechanisms. While in CCl_4_-treated livers the downregulation of the components of elastic fibers and microfibrils was anticipated to give rise to the increased ECM stiffness; in DDC-treated livers the decrease in col6 that participates in the formation of microfibrils and the increase in col1 and fibronectin content were likely playing a role^35^,^36^. Increased stiffness of the ECM has been shown to promote cell migration and proliferation of hematopoietic stem and progenitor cells^37^,^38^, thus, it can be envisaged that the changes in molecular composition of the ECM, potentially in conjunction with the resulting alterations in the mechanical properties promote the cell proliferation and migration also in the regenerating liver (Fig. 7). The potential role of ECM structural components in modulating cell proliferation and thereby liver repair was further corroborated by our findings that demonstrated selective growth stimulation properties for ECM components. For example, while col4 was most efficient in supporting hepatocyte proliferation *in vitro,* the high col4 expression coincided with proliferating hepatocytes also in regenerating liver suggesting a scenario where the presence of col4 sustains the proliferation of uninjured hepatocytes while it is downregulated in areas where the extent of tissue damage requires extensive ECM remodeling. Concordantly, fibronectin and col1, which were upregulated in DDC-treated livers, supported the proliferation of ck19-expressing cholangiocyte-like cells in culture, as bile ducts are primarily targeted in this liver damage model. Since the amount of fibronectin was increased in both types of liver damage it might play a universal role in liver repair as it has been shown previously that fibronectin promotes the differentiation of liver progenitor cells towards the hepatocyte phenotype^39^.

In addition to structural proteins the levels of several non-structural ECM components were significantly altered in livers subjected to damage. Interestingly, we found that the expression of vitronectin and Olfm4, which were highly upregulated in CCl_4_-treated livers, was limited to pericentral hepatocytes. Vitronectin and Olfm4 act as important regulators of cell adhesion and migration in tissue regeneration and cancer progression^17^,^18^. Since Olfm4 specifically stimulated hepatocyte proliferation *in vitro*, it is likely that its upregulation in damaged tissue portions is necessary to stimulate the migration of proliferating periportal hepatocytes to the site of damage and sustain their proliferation there. Thbs4 is a glycoprotein with proangiogenic properties that sustains tissue remodeling and regeneration^20^,^40^,^41^. Since Thbs4 stimulated more efficiently NHC growth *in vitro*, a strong Thbs4 expression around the portal area in damaged livers is anticipated to promote liver regeneration via activation of portal NHCs. Armcx2 is involved in development and in the maintenance of tissue integrity^19^. We demonstrated that Armcx2 was more efficient in stimulating NHC proliferation *in vitro* but it was highly expressed in damaged mouse and human liver parenchyma suggesting the presence of a potential Armcx2-mediated growth stimulation of NHCs by hepatocytes. Alternatively, it might participate in controlling the hepatocyte proliferation during regeneration since Armcx2 may also act as a limiting factor for cell proliferation as it is lost in many cancers^19^. The results of the current work and their potential relevance to liver repair are summarized on Fig. 7.

Although in this work we noted several similarities between the damaged mouse and human liver samples in the ECM protein expression pattern, one has to keep in mind, though, that mouse and human samples were collected from livers subjected to different tissue damage modalities. In the current work mice were treated with a short-term impact of a damaging factor that induced physiological tissue repair whereas in the case of human samples ongoing pathological impact (eg alcohol, hepatitis C infection) had caused a long-term tissue remodeling response and in severely damaged cases-fibrosis. Nevertheless, it is tempting to envisage that the similarities identified between the human and mouse samples are not purely coincidental and are suggestive of a general stress response pattern of mammalian liver ECM.

Taken together, we systematically analyzed the changes in ECM that took place during liver tissue repair and identified major alterations in the liver ECM structure and mechanical properties potentially arising as a consequence of the alterations in its composition. In addition we identified a number of previously uncharacterized liver ECM proteins and studied their potential role in regulating the proliferation of liver cells.

The general pattern of ECM alterations revealed a well-orchestrated multi-level response in the ECM during liver regeneration.

## Additional Information

**How to cite this article**: Klaas, M. *et al.* The alterations in the extracellular matrix composition guide the repair of damaged liver tissue. *Sci. Rep.* **6**, 27398; doi: 10.1038/srep27398 (2016).

## Supplementary Material

Supplementary Information

Supplementary Information
