# Supplementary material for: The alterations in the extracellular matrix composition guide the repair of damaged liver tissue
Source: Sci Rep. 2016 Jun 6;6:27398. doi: 10.1038/srep27398 (PMC4893701; doi:10.1038/srep27398)
Supplement: Supplementary Information [file srep27398-s1.pdf]

The alterations in the extracellular matrix composition guide the repair of damaged liver tissue

Mariliis Klaas[1], Triin Kangur[2], Janeli Viil[1], Kristina Mäemets-Allas[1], Ave Minajeva[3,4], Krista Vadi[1], Mikk Antsov[2], Natalia Lapidus[4], Martin Järvekülg[2] and Viljar Jaks[1,5,*]

[1] Department of Cell Biology, Institute of Molecular and Cell Biology, University of Tartu, Estonia

[2] Laboratory of Physics of Nano Structures, Institute of Physics, University of Tartu, Estonia

[3] Department of Pathological Anatomy and Forensic Medicine, Faculty of Medicine, University of Tartu, Estonia

[4] Department of Pathology, Clinic of Diagnostics, West Tallinn Central Hospital, Estonia

[5] Department of Biosciences, Karolinska Institutet, Stockholm, Sweden

Supplementary Methods

Mass-spectrometry analysis

Decellularized liver samples (n=3) were homogenized in lysis buffer containing 7 M urea, 4% CHAPS, 10 mM DTT and 40 mM Tris-HCl (pH 7.5) using Precellys 24 homogenizer and CK14 Precellys lysing kit (both Bertin Technologies, Paris, France) at 5500 rpm for 3 cycles of 30 s. Proteins were purified by methanol/chloroform protein precipitation. Protein reduction and alkylation was performed in a solution containing 7 M urea, 2 M thiourea, 100 mM ammonium bicarbonate by adding 2.5 mM DTT and 5 mM iodoacetamide for 30 min, at room temperature. Protein digestion was performed by Lys-C protease (Life Technologies) treatment (200 ng per reaction) 4h. The solution was then diluted 5x with 100 mM ammonium bicarbonate solution and after adding 200 ng trypsin (Life Technologies) the samples were incubated for 16 h. The digestion was stopped by adding triflouroacetic acid to a concentration of 1.0 %. The peptides were purified by C18 StageTips (Thermo Scientific, Wien, Austria). Peptides were separated on Agilent 1200 series nano-LC with in-house packed (3 µm ReproSil-Pur C18AQ particles, Dr. Maisch GmbH, Ammerbuch-Entringen, Germany) 15 cm 75 µm ID emitter-columns (New Objective, Woburn, MA) using a 240 min 8-40% B gradient where buffer A was 0.5% acetic acid in water and B, 0.5% acetic acid in 80% acetonitrile. Separated peptides were eluted at 200 nl/min (spray voltage 2.0-2.2 kV) to a LTQ Orbitrap XL (Thermo Fisher Scientific, San Jose, CA) mass-spectrometer operating with a top-5 MS/MS strategy with a minimum of 1 s cycle time. Dynamic exclusion was set to 110 s. Only charge states over +1 were analysed. Raw data were identified and quantified with

MaxQuant 1.4.0.8 software package. Search was performed against UniProt (www.uniprot.org) database using the tryptic digestion rule.

Calculation of the indentation modulus by applying the Hertzian contact mechanics model

For this model, the force-displacement curve of an indentation of a flat surface with an elastic half-space is governed by the following equation:

$$F = \frac{4}{3}E^{*}R^{\frac{1}{2}}d^{\frac{3}{2}} \quad (1)$$

where F is the applied force, $E^{*}$is the reduced elastic modulus, R the radius of the indenter and d the indentation depth. The reduced elastic modulus can be calculated via the following equation $\frac{1}{E^{*}} = \frac{1-\nu_1^2}{E_1} + \frac{1-\nu_2^2}{E_2}$, where E_1, $E_2$and ν_1, ν_2 are the elastic moduli and Poisson ratios of the bodies in contact, respectively. As the biological sample is much softer than the stiff silicon AFM tip the deformation of the tip can be neglected and the reduced elastic modulus can be replaced with the elastic stiffness of the surface. Each force-displacement curve was individually fitted by eq. (1), where the elastic stiffness $E^{*}$was varied.

Generation of Armcx2, Olfm4 and Thbs4 expression vectors

To generate Armcx2, Olfm4 and Thbs4 proteins expressing plasmids (pcDNA3.1Zeo_Armcx2_Cd33SP, pcDNA3.1Zeo_Olfm4_Cd33SP and pcDNA3.1Zeo_Thbs4), the cDNA of the genes was amplified by PCR using commercially available cDNA sequences from BioCat GmbH (Heidelberg, Germany) and inserted into pcDNAZeo3.1 vector. To ensure the secretion of the selected proteins into the cell media, CD33 secretion signal (MPLLLLLPLLWAGALA) was

fused with the N-terminus of the Armcx2 and Olfm4 sequences (Thbs4 has its own strong secretion signal). All constructs were verified by sequencing.

Cell transfections

Human embryonic kidney cells HEK293 (obtained from the American Type Culture Collection) were cultivated in IMDM medium (Lonza, Basel, Switzerland) supplemented with 10% (v/v) fetal bovine serum, 50 units/ml penicillin, 50 µg/ml streptomycin and 1 mM L-glutamine (full media). Prior transfection the full media was replaced with IMDM containing 1X Insulin-Transferrin-Selenium supplement (Life Technologies, Warrington, UK). HEK293 cells were transfected with 6 µg pcDNA3.1Zeo_Armcx2_Cd33SP, pcDNA3.1Zeo_Olfm4_Cd33SP and pcDNA3.1Zeo_Thbs4 plasmids by using TurboFect reagent (Thermo Scientific) according to manufacturer´s instructions. 72 hours after transfection the cells and cell media were collected for protein expression analysis and for further use in liver cell culture experiments.

Western Blot

HEK293 cells transfected with 6 µg pcDNA3.1Zeo_Armcx2_Cd33SP, pcDNA3.1Zeo_Olfm4_Cd33SP and pcDNA3.1Zeo_Thbs4 vectors were lysed in NP40 buffer 72 hours after transfection. For secreted proteins analysis, cell media was inserted into dialysis bags (Spectrum Medical Industries, Houston, TX) and incubated at constant stirring overnight at 4 °C in 50 mM Tris pH7.5 buffer. Secreted proteins from cell media were precipitated with 5 volumes of ice cold acetone followed by 15 min centrifugation at 4 °C 15000 rpm. Precipitate was dissolved in 4x Laemmli buffer. The lysates were separated using SDS-PAGE (10% gel) and transferred to polyvinylidene difluoride (PVDF) membrane (Thermo Scientific). The

PVDF membrane was blocked with 5% non-fat dry milk (AppliChem, Darmstadt, Germany) at room temperature for 1 hour, followed by incubation with the primary antibody at 4°C overnight (Supplementary Table S2). Subsequently, the membrane was washed with PBS buffer 3x10 minutes and incubated with the corresponding biotin/streptavidin alkaline phosphatase conjugate antibodies for 1 hour at room temperature. The signals were visualized by adding alkaline phosphatase substrate to the membrane. The expression of Armcx2, Olfm4 and Thbs4 recombinant proteins in the conditioned medium can be seen in Supplementary Fig. S1.

Mouse liver cell proliferation analysis

Acute liver injury was induced by intraperitoneal injection of CCl_4 (1 ml/kg) in sunflower oil and mice (n=3) were sacrificed after 2 or 6 days. Alternatively, mice were fed with 0.1% DDC-supplemented diet for 1, 2 or 5 weeks. At specified timepoints the mice were sacrificed and the livers frozen for cryosectioning. Tissue sections were stained for Ki67-antigen expression to estimate cell proliferation. At least 5 different fields of view were examined for each sample containing approximately 2000 cells each.

Supplementary Tables

Supplementary Table S1. Antibodies used in immunofluorescence studies

Antibody (host)	Dilution	Manufacturer; catalog number
Armcx2 (rabbit pAb)	1:100	antibodies-online GmbH, Aachen, Germany; ABIN484589
Chicken anti goat IgG pAb, Alexa 647 conjugated	1:1000	Life Technologies, Grand Island, NY; A-21469
Chicken anti mouse IgG pAb, Alexa 647 conjugated	1:1000	Life Technologies, Grand Island, NY; A-21463
Chicken anti rabbit IgG pAb, Alexa 488 conjugated	1:1000	Life Technologies, Grand Island, NY; A-21441
ck19 (mouse mAb)	1:40	Novus Biologicals, Littleton, CO; (clone: BA17)
ck19 (rabbit pAb)	1:10000	Abcam; Cambridge, UK; MAB3506
ck19 (rat mAb supernatant)	1:1	Developmental Studies Hybridoma Bank, Iowa City, IA; (clone: troomaIIIg)
Collagen type I (rabbit pAb)	1:200	Abcam, Cambridge, UK; ab34710
Collagen type IV (rabbit pAb)	1:200	Abcam, Cambridge, UK; ab6586
Collagen type V (rabbit pAb)	1:200	antibodies-online GmbH, Aachen, Germany; ABIN669996
Donkey anti goat IgG pAb, Alexa 488 conjugated	1:1000	Life Technologies, Grand Island, NY; A-11055
Donkey anti rabbit IgG pAb, Alexa 647 conjugated	1:1000	Life Technologies, Grand Island, NY; A-31573

Donkey anti rat IgG pAb, Alexa 594 conjugated	1:1000	Life Technologies, Grand Island, NY; A-21209
Elastin (rabbit pAb)	1:100	Abcam, Cambridge, UK; ab21610
Fibronectin (rabbit pAb)	1:200	Abcam, Cambridge, UK; ab2413
Goat anti rat IgG pAb, Alexa 488 conjugated	1:1000	Life Technologies, Grand Island, NY; A-11006
HNF4α (goat pAb)	1:200	Santa Cruz Biotechnology, Santa Cruz, CA; sc-6556
Ki67 (rabbit mAb)	1:100	Spring Bioscience, Pleasanton, CA; M3060 (clone: SP6)
Ki67 (rat mAb)	1:200	eBioscience, San Diego, CA; 14-5698 (clone: SolA15)
Major urinary protein (MUP)	1:200	Nordic-MUbio, Susteren, The Netherlands; GAM/MUP
Olfm4 (rabbit pAb)	1:100	Cloud-Clone Corp., Houston, TX; PAA162Mu01
Olfm4 (rabbit pAb)	1:100	Novus Biologicals, Littleton, CO; NBP2-24535
Thrombospondin-4 (goat pAb)	1:100	R&D Systems, Abingdon, UK; AF2390
Vitronectin (rabbit pAb)	1:50	antibodies-online GmbH, Aachen, Germany; ABIN675518
Vitronectin (rat mAb)	1:50	R&D Systems, Abingdon, UK; MAB38751 (clone 347317)

Supplementary Table S2. Antibodies used in Western blot

Antibody (host)	Dilution	Manufacturer; catalog number
Armcx2 (rabbit pAb)	1:1000	Proteintech, Manchester, UK; 12200-1-AP
Olfm4 (rabbit pAb)	1:1000	Novus Biologicals, Littleton, CO; NBP2-24535
Thrombospondin-4 (goat pAb)	1:1000	R&D Systems, Abingdon, UK; AF2390
Polyclonal goat anti-rabbit, biotinylated	1:3000	DAKO, Glostrup, Denmark; E0432
Polyclonal rabbit anti-goat, biotinylated	1:5000	DAKO, Glostrup, Denmark; E0466
Streptavidin/alkaline phosphatase	1:3000	DAKO, Glostrup, Denmark; D0396

Supplementary Figures

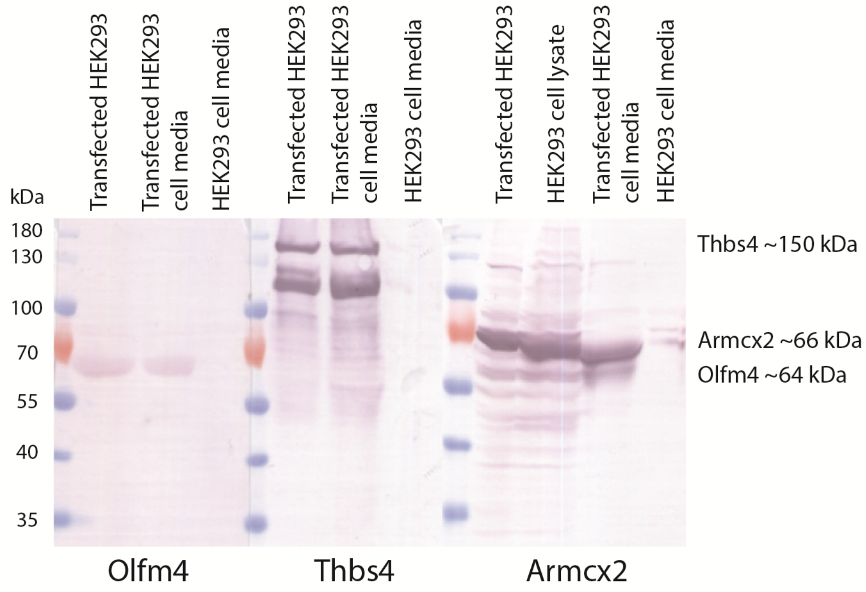

Supplementary Figure S1. Expression of secreted Olfm4, Thbs4 and Armcx2 proteins in transfected HEK293-conditioned media.

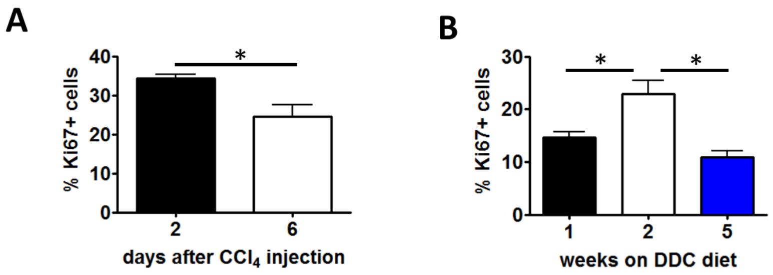

Supplementary Figure S2. Liver cell proliferativity after CCl_4 injection (A) and on DDC diet (B). The percentage of proliferating cells ± SD is shown (n=3). * indicates a statistically significant (P<0.01) difference.

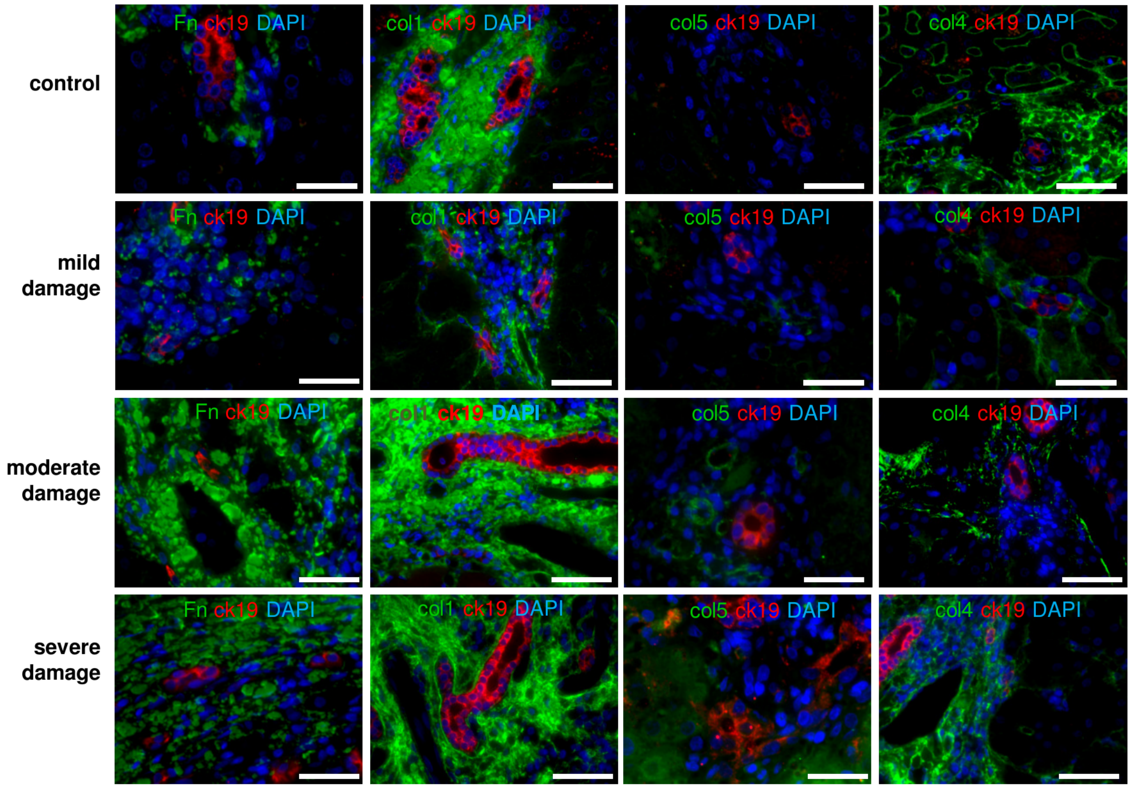

Supplementary Figure S3. Immunofluorescence microscopy analysis of structural ECM components altered in damaged human (n=5) livers. Fn - fibronectin. Scale bars 50 μm.

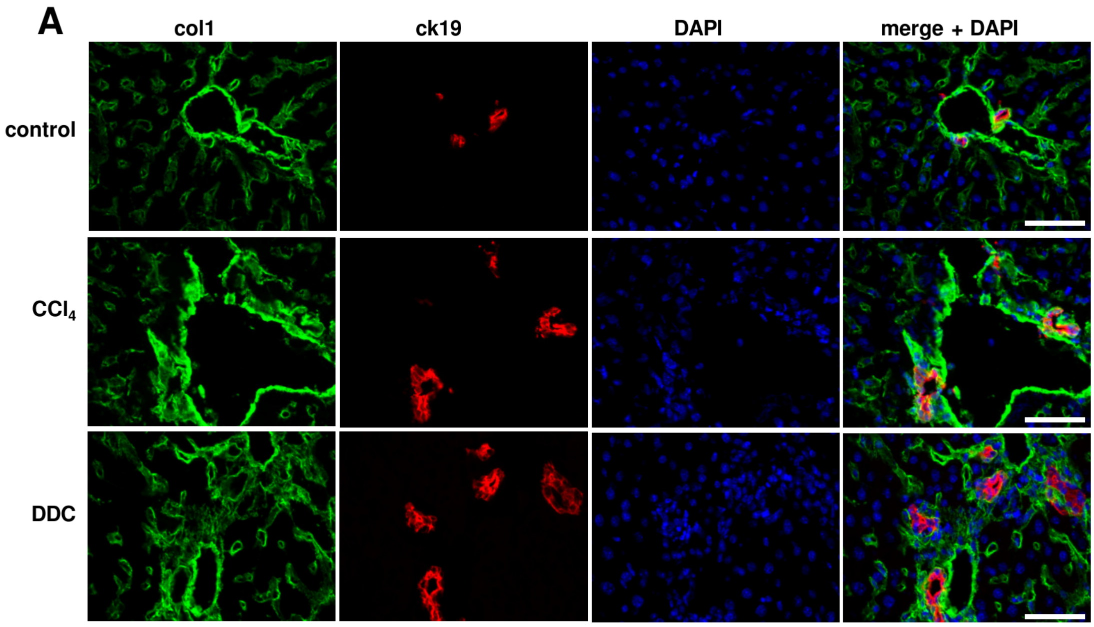

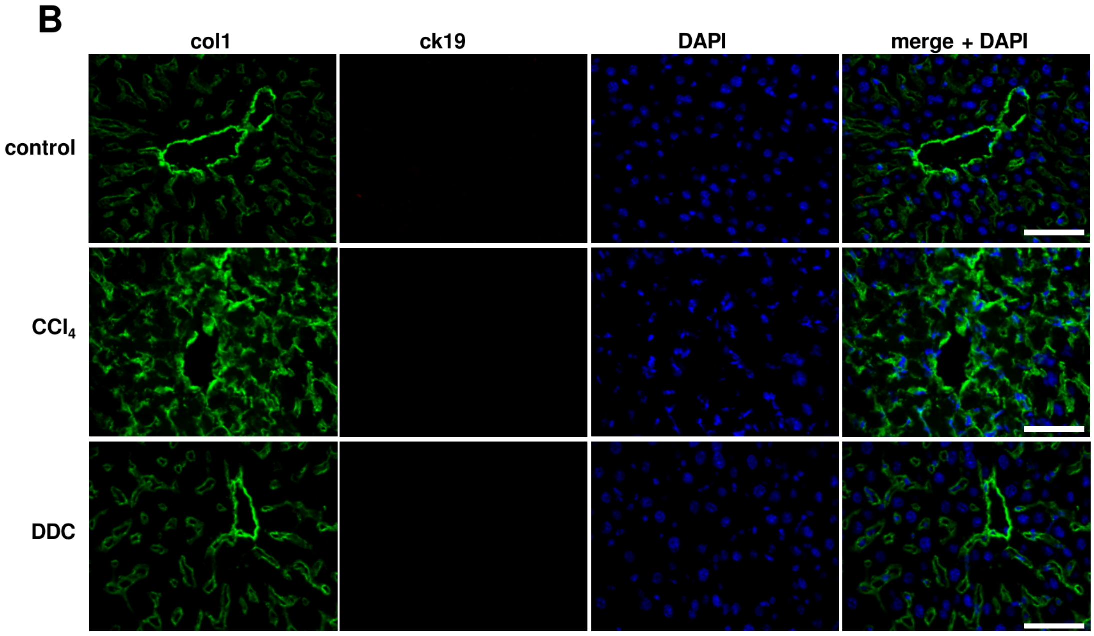

Supplementary Figure S4. Immunofluorescence microscopy study of col1 expression in damage in mouse liver periportal (A) and pericentral (B) area, n=4. Scale bars are 50 μm in figure A and 200 μm in figure B.

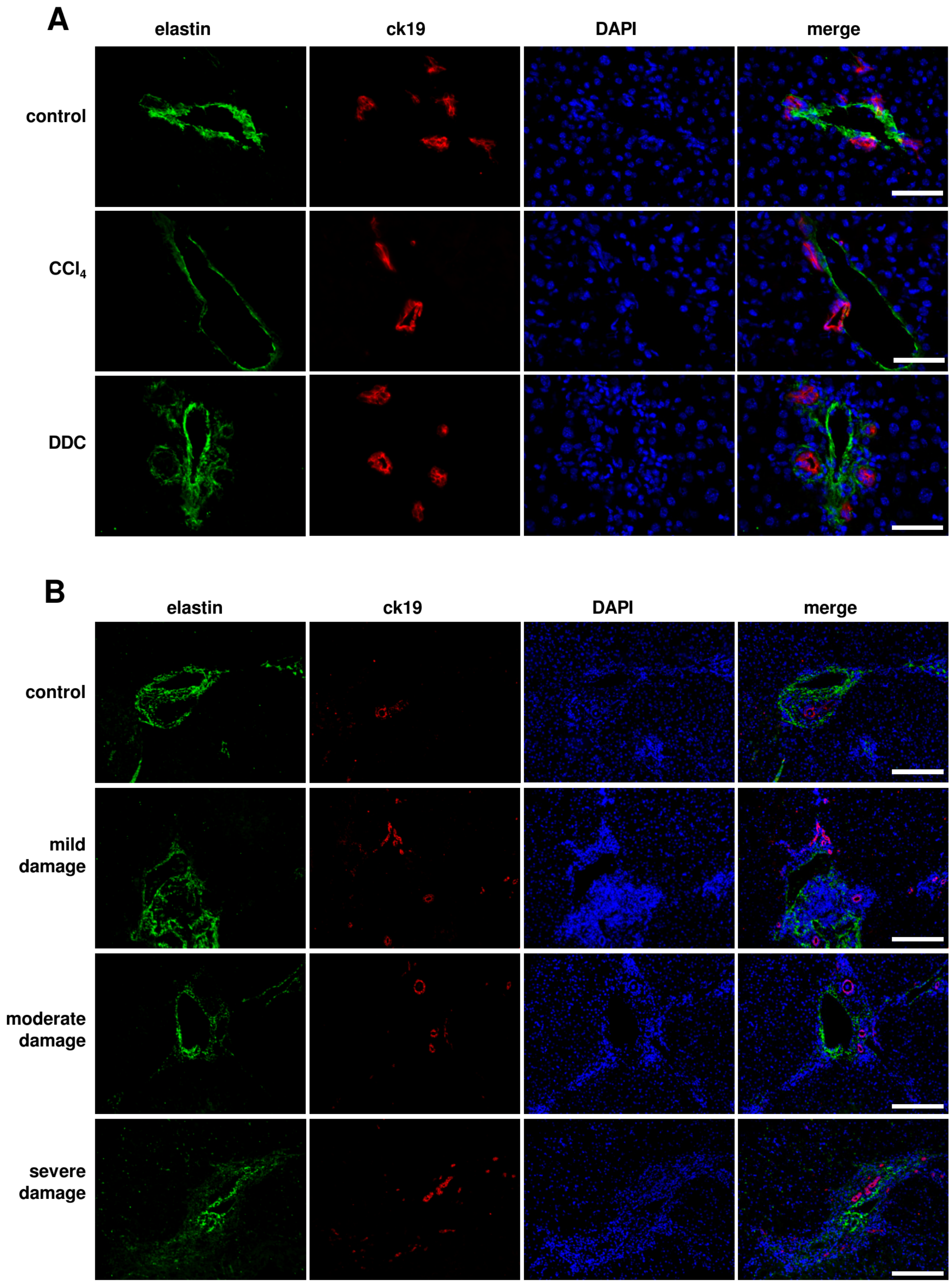

Supplementary Figure S5. Immunofluorescence microscopy study of elastin expression in damage in mouse (A, n=4) and human (B, n=5) livers. Scale bars are 50 µm in figure A and 200 µm in figure B.

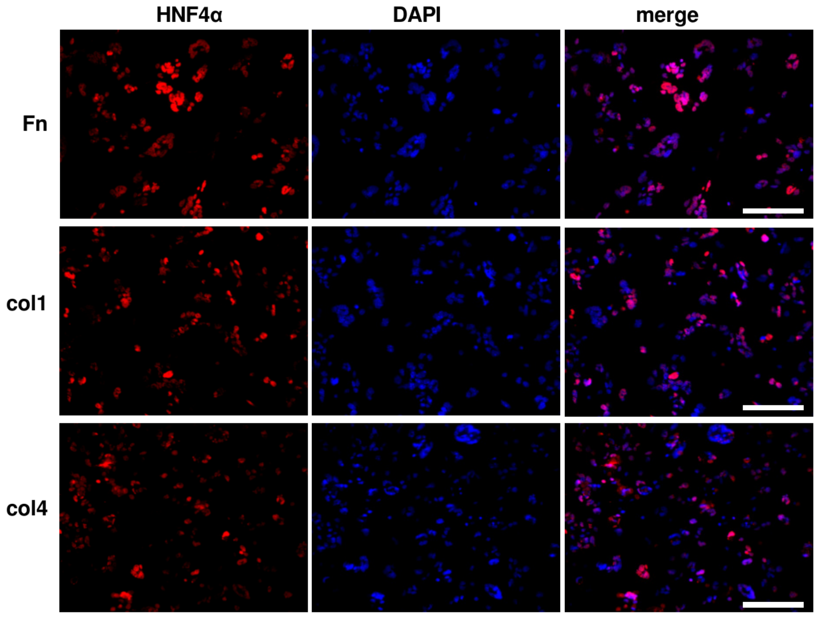

Supplementary Figure S6. Hepatocyte fraction cell growth assay on dishes coated with fibronectin (Fn), col1 or col4. Cells stained with HNF4α antibody. Scale bars are 200 μm.

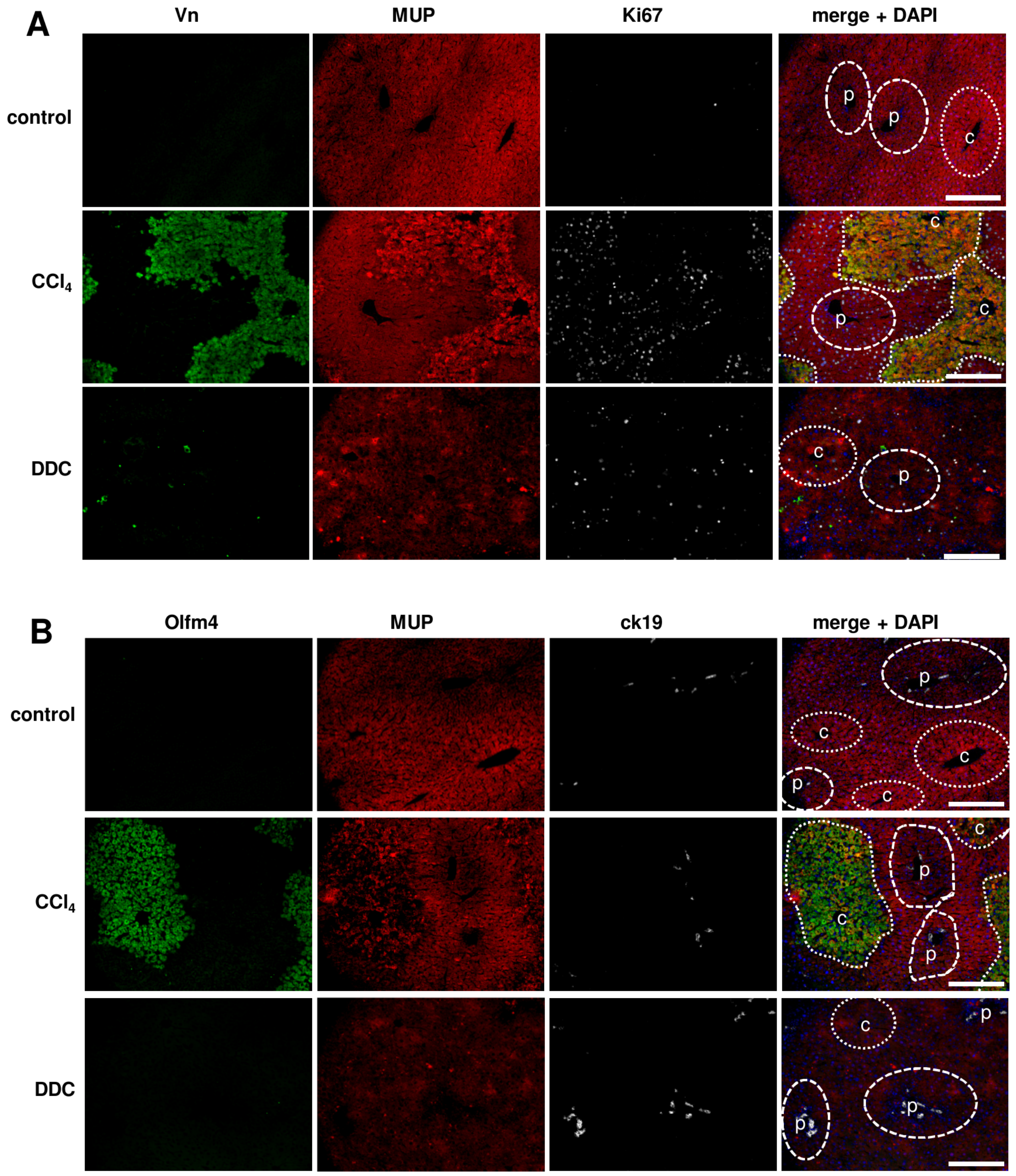

Supplementary Figure S7. Immunofluorescence microscopy study of Vn+ (A) and Olfm4+ (B) cell population in mouse liver damage. Scale bars are 200 μm. C indicates to pericentral area, p indicates to periportal area.

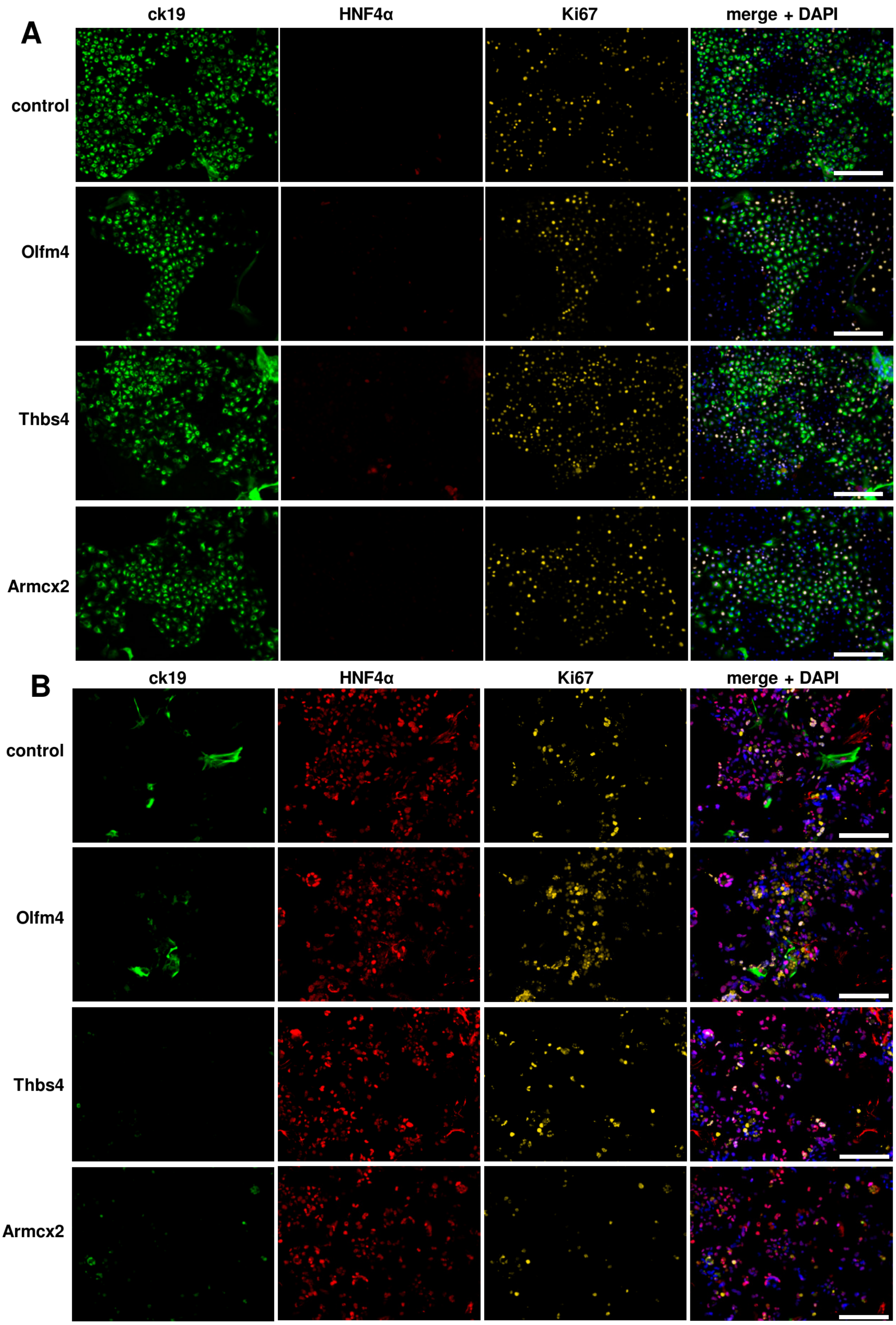

Supplementary Figure S8. Non-hepatocyte (A) and hepatocyte (B) cell fraction growth assays (n=3) on dishes coated with col1 and added HEK293-conditioned media containing Olfm4, Thbs4 or Armcx2 recombinant protein. Media from sham-transfected cells was used as a control. Scale bars are 200 μm.
